# Navigating dry eye relief: Meibo’s approach to controlling tear evaporation

**DOI:** 10.1097/MS9.0000000000002550

**Published:** 2024-09-10

**Authors:** Bisma Ahmed, Yumna Shahzad, Wajiha Urooj, Amal Siddiqui, Zaib un Nisa Mughal, Khabab Abbasher Hussien Mohamed Ahmed

**Affiliations:** a Department of Medicine, Jinnah Sindh Medical University, Karachi, Pakistan; b Faculty of Medicine, University of Khartoum, Khartoum, Sudan

## Unveiling dry eye disease: an overview

As a multifactorial ocular surface condition, dry eye was redefined in 2017 by the Tear Film and Ocular Surface Society (TFOS) during the Dry Eye Workshop II (DEWS). The absence of tear film homoeostasis is a characteristic that presents clinically as discomfort, a foreign body gritty sensation, hyperaemia, and blurred vision. Corneal ulcers may also present in severe, unmanaged cases, compromising visual acuity. Multiple essential etiologies behind DED include unstable and hyperosmolar tear film, inflamed and injured eye surface, and neurosensory abnormalities^1,2^.

A common ocular surface ailment, dry eye disease (DED) has two main subtypes: evaporative DED, which is caused by excessive evaporation of the tear film, and aqueous-deficient DED, which is characterized by diminished lacrimal production. These DED subtypes have different prevalence rates. Aqueous-deficient DED alone is thought to occur in only 10–15% of DED cases, according to estimates. In fact, meibomian gland dysfunction (MGD) is the main cause of evaporative DED, which accounts for the great majority of DED cases that are evaporative in character or contain an evaporative component^3^.

In dry eye illness, the subjective symptoms are frequently nonspecific. Redness, burning, tingling, feeling like a foreign body, itching, and photophobia are a few of them. Dry eye symptoms often include somewhat prominent conjunctival redness and ocular surface damage with punctate epithelial erosions (superficial punctate keratitis); temporal conjunctival folds parallel to the lid edge are suggestive^4^.

Variations in the severity of the condition depend on known risk factors: females, increasing age, an arid environment, smoking, digital device use, and staying up late at night. Also, individuals with anxiety, stress, or depression are more likely to experience dry eyes^5,6^.

## The hidden mechanics: pathophysiology of DED

Over the past 20 years, there has been a significant advancement in our understanding of the pathophysiology of dry eye, and this progress is still ongoing. Increased tear osmolarity (diffusely or in the area of tear breakup) is associated with tear instability. This increases the production of innate inflammatory molecules and activates stress signalling pathways in resident immune cells and the ocular surface epithelium. This starts a vicious cycle that can worsen symptoms and further impair tear function. It is frequently difficult to identify a single cause for the majority of cases of dry eye disease, which highlights the significance of addressing all modifiable risk factors. Extrinsic factors that can contribute to this inflammatory cycle include exposure and desiccating environments. Intrinsic factors that can contribute to this cycle include ageing, autoimmunity, and drying medications^7^. Normally, the central nervous system, which controls the production of tears and blinking, receives impulses from the stimulation of nerve terminals in the cornea and nasal passages. Additionally, the lachrymal gland is encouraged to create the watery portion of the tear film by activating the nerve end. In order to form a mucin layer, goblet cells in the conjunctiva and ocular surface are finally accelerated. The two main characteristics of this condition are thought to be ocular inflammation and hyperosmolarity of the tear film. The generation of inflammatory mediators, such as interleukin 1 and tumour necrosis factor-α, into the ocular surface due to hyperosmolarity damages both the goblet cells and the ocular epithelium, causing disruption in the tear film and eventual DED. It is unknown, therefore, if the tear film’s hyperosmolarity develops early in the DED or if it is a sign of tear film dysfunction^8^.

## Gauging the glare: assessing dry eye severity

Three common instruments are used to assess the severity of DED: the Dry Eye Questionnaire (DEQ-5), the Standardised Patient Evaluation of Eye Dryness (SPEED), and the Ocular Surface Disease Index (OSDI). It is followed by a comprehensive eye exam and utilization of in-office devices (slit-lamp exam, meibography) to assess the viability of tear film and quantify the tear volume. Fluorescein or lissamine green stain further aids in illuminating abnormalities consistent with dry eye. The tear film breakup time (TBUT) test measures the amount of time it takes for tears in an eye stained with fluorescein to separate after blinking; a time less than 10 s is considered abnormal and may indicate tear film instability. However, the Schirmer test assesses tear generation by soaking a paper strip. A reading of 5 mm or less is considered abnormal. Lastly, measurement of MMP-9 (matrix metalloproteinase 9), an inflammatory marker, is markedly elevated in DED patients. In any case, in customary clinical settings, there is currently no “gold standard” or adequate measure of tests for diagnosing itchy, dry eyes^1,9^.

## Legacy solutions: a retrospective on traditional DED treatments

The primary treatment is to restore the visual surface and tear film’s equilibrium by disrupting the recurrent pattern of DED. DED, linked to MGD, is often treated in a progressive approach, starting at home with over-the-counter (OTC) ocular lubricants and interventions, then moving on to medical supplies and corporate devices^9^.

Initially, house-care practices are recommended with eyelid cleaning twice daily, together with warm packs that increase the liquefaction and flow of meibum, improving tear film stability. Due to the long-term commitment, a lack of patient compliance was noted in effectiveness^9,10^. The supplementation of essential fatty acids, omega-3 and anti-inflammatory omega-6 (gamma linoleic), improved the OSDI score and sustained ocular comfort. However, their efficacy in treating DED still needs to be determined.

OTC lubricating ocular drops are a first-line therapeutic option commonly accessible in a number of lipid- and water-based preparations. A comparison of the two in the randomized trial depicted the latter’s efficacy, precisely in evaporative DED^1,9,10^. Rebamipide, an oral mucin secretagogues, and Lactoferrin, an enteric-coated tablet, are also used to treat DED^4^.

In 2003, the FDA approved a fungal antimetabolite, topical cyclosporine A (CsA) 0.05% emulsion, for dry eye. It improves symptoms and OSDI scores and increases tear production. However, its cost restricts its application among other adverse features like conjunctival hyperaemia, epiphora, irritation, foreign body sensation, and visual haziness. Subsequently, in July 2016, Lifitegrast 5% attained the FDA approval. It works on dry eye signs and systemic effects without fundamental immunosuppression or nearby degeneration. However, it induces instillation-site redness, discomfort, sensitivity, decreased visual clarity, and dysgeusia^10^.

An ophthalmic suspension of corticosteroid [loteprednol etabonate (LE) 0.25%] was also approved for treating DED (for 14 days)^9^. Oral tetracyclin (40–400 mg/day for doxycycline) in small oral dosages further treats corneal ulceration; nonetheless, large doses cause skin and gastrointestinal symptoms. 1% azithromycin eye drops for treating blepharitis induced by MGD further restore tear film viability^4^.

Office-based therapeutic interventions are frequently utilized as second-line treatments. Microblepharo exfoliation, laser therapy, intraductal examination, and heat stimulation equipment (e.g. LipiFlow, iLux, TearCare, MiBo ThermoFlo) of the meibomian glands, and manual stimulation of the meibomian glands are all options. The trials assessing manual expression conclude with symptoms, OSDI, TFBUT, and meibum quality improvements^9,10^.

Microblepharo exfoliation devices eliminate bacterial biofilm from the eyelids and clean them. On the other hand, heat stimulation gadgets use pressure and warmth to break down and release meibum. Various trials evaluated the different available thermal devices and found a comparable safety and efficacy profile with no device-related adverse severe outcomes.

Intense pulsed light (IPL) was approved by the US Food and Drug Administration (FDA) to cure DED caused by MGD. Though its mechanism is poorly understood, it is hypothesized that temperature variations may alter lipids and cytokines in the tear film. The only barrier to these treatments is their cost, as most insurance plans do not cover them^1,9,10^.

Lastly, the office-based approach is a relatively invasive intraductal probing of the meibomian gland. It mechanically opens the gland orifice, relieving the obstructive MGD and improving lipid titres and meibum viscidity^9^.

Specific devices, nasal sprays, are also helpful for patients with watery tears as intra- or additional nasal neurostimulators, which were FDA-approved. At the same time, scleral focal points can be utilized as a liquid-filled supply for a patient with an irregular corneal surface. Although ITN can induce nasal distress, nasal bleeding, burning, irritation, intermittent electrical discomfort, nasal clogs, face pain, and migraines and cannot be utilized in patients with heart pacemakers or other implants. The extended and expensive fitting cycle is one drawback of using scleral focal points for DED^4,10^.

Autologous serum drops improve dry eye disease symptoms and objective signs. Newer products, like eye-platelet-rich plasma and growth factor plasma, aim to maximize growth factor concentration but must be authorized by the FDA, making access limited and often unaffordable.

The amniotic film is validated for different ocular problems and can be utilized for the DED as it has calming and soothing properties^4^. ProKeraTM is accessible as a gadget that can be put on a patient’s eye in the workplace and has a ring that holds the amniotic membrane transplant (AMT) setup^10^. Other surgical procedures include occlusion of lachrymal puncta, tarsorrhaphy, and transplantation of salivary glands^2^.

## Spotlight on Meibo: a breakthrough in dry eye therapy

The US Food and Drug Administration (FDA) recently approved NOV03 (MIEBO [perfluorohexyloctane ophthalmic solution]), a novel topical medication therapy, to treat the signs and symptoms of DED. It is an ophthalmic drop that is single-entity, water-free, and preservative-free and contains PFHO, or perfluorohexyloctane^11^. Because PFHO is a semifluorinated alkane with six perfluorinated and eight hydrogenated carbons, it is physiologically inert. Because of this, it possesses amphiphilic qualities, which enable it to form a monolayer when applied topically at the tear film air-liquid interface. When the temperature rises above –7.1 °C, PFHO becomes a liquid and does neither absorb nor scatter visible light. Its refractive index is comparable to that of water; hence it causes negligible blurriness in eyesight^12^.

## Mechanistic insights: the science behind the relief

Miebo concentrates on evaporating tears rather than creating new ones^11,13^. Because PFHO forms a liquid layer at the tear film’s air-liquid interface, effectively blocking evaporation, it may enable important functions of meibum. Moreover, the bipartite molecular structure of PFHO confers a decreased surface tension, hence expediting its diffusion throughout the ocular surface^11,12,14^. This spreading characteristic also helps to reduce friction, which in turn helps to lessen pain when blinking. Drop overflow following instillation may be lessened by PFHO’s ability to produce smaller drop sizes than aqueous drops due to its lower surface tension^14^.

## Pharmacokinetic pathways: Meibo in action

Restricted absorption was found in a pharmacokinetic study on the systemic availability of PFHO following topical application to the eye’s surface. It has been demonstrated in animal experimental models that most of the PFHO that is delivered to the eye evaporates into the atmosphere from the ocular area. Any minute amount that enters the digestive system from the drainage of the tear duct is eliminated in the form of faeces within a day. Exhalation is the means by which the fractional PFHO that is absorbed systemically is released. According to an environmental analysis done for US FDA approval, there would be less than 1 part per billion of PFHO in the aquatic environment at the point of entry. This indicates that PFHO’s effects on the environment are likely negligible. With a 62% greater O2 carrying capacity than air, PFHO facilitates O2 transfer to the ocular surface, facilitating wound healing and preventing corneal oedema while not serving as a barrier^14^.

## Clinical triumphs: efficacy of Meibo in trials

Two phase 2 randomised controlled studies involved the recruitment of patients with DED and MGD (SEECASE) and one phase 3 randomised controlled research (GOBI) to show the efficacy and safety of NOV03. Given twice or four times a day, NOV03 demonstrated a significantly larger reduction in DED signs and symptoms and a better safety and tolerability profile when compared to the isotonic saline (0.9%) control in SEECASE. The Eye Dryness Score showed statistically significant differences at week eight (*P* = 0.002 for BID and *P* < 0.001 for QID) between the baseline and control groups^15,16^.

## Safety lens: scrutinizing Meibo’s safety profile

Eye dryness and Total corneal fluorescein staining [tCFS], measured on a visual analogue scale [VAS], the two main indications and symptoms of DED, also showed statistically significant decreases when NOV03 was dosed four times a day compared to hypotonic saline (6%) in GOBI, the phase 3 clinical trial. Results of a second phase 3 trial with a similar design, called MOJAVE, which assessed NOV03’s safety and effectiveness in adults with DED linked to MGD. For 8 weeks, patients were randomly assigned to inject 1 drop of study medicine or saline (0.6%) into both eyes four times a day. The treatment assignment was concealed from both patients and investigators. In comparison to patients receiving the saline control medication, patients treated with NOV03 showed a substantially higher reduction from baseline at week 8 in both the tCFS score and the VAS dryness score, so fulfilling both primary efficacy endpoints^11,15^.

A randomized, controlled phase 2 study (SEECASE), two randomized, controlled phase 3 clinical trials (GOBI and MOJAVE), and an open-label safety extension study (KALAHARI) involving a subset of patients from the GOBI study all contributed to the FDA’s approval of PFHO ophthalmic solution. All of these trials showed that PFHO is safe and related to fewer symptoms and indications of dry eyes^14^.

No absolute contraindications currently exist for use of perfluorohexyloctane for DED. However, perfluorohexyloctane has not been studied in pregnant, breastfeeding, or paediatric populations^11^. Safety and efficacy in patients younger than 18 years have not been established.

The most common eye side effect seen in studies was blurred vision (1–3% of patients reported blurred vision and eye redness)^17^. The adverse effects in the NOV03 population were mild to moderate, including blepharitis, conjunctival hyperaemia, conjunctival papillae, ocular hyperaemia, obscured vision, hordeolum (eye cyst), and a decrease in visual acuity. No non-ocular side effects were attributed to the NOV03 treatment^11^.

## Practical insights: using Meibo in daily life

Miebo is supplied in a multiple-dose 5 ml bottle; it can be stored at room temperature and used until the expiration date on the bottle. The recommended dosage of Miebo is 1 drop 4 times daily into the affected eye(s). Patients who wear contact lenses should remove them prior to and for at least 30 minutes after administering Miebo^18^.

## Comparative review: Meibo vs. traditional therapies and future perspectives

Perfluorohexyloctane, the active ingredient in MIEBO, is specially formulated to address the evaporative aspect of dry eye disease (DED) unlike other treatment options; however, it is also beneficial to other patients with the condition who may need to stabilize their tear film and reduce aqueous tear evaporation. MIEBO is different from other topical eye medicines because its composition is free of water and preservatives, allowing the molecule to act itself as a therapeutic agent. Because of this, the molecule’s ability to modify its pH without the need for modulation results in enhanced comfort during drop instillation. Unlike most other eye drops, this one feels silky and smooth upon contact, indicating that it has the capacity to create a long-lasting coating on the ocular surface that may stop tears from evaporating^19^. Microbial development is not feasible in MIEBO since it is a non-aqueous liquid that contains 100% perfluorohexyloctane^20^.

Perfluorohexyloctane ophthalmic solution is the first and only DED medication authorized by the FDA that targets tear evaporation directly rather than tear production. An ongoing cycle of irritation and friction is set off by excessive tear evaporation, which damages the surface of the eyes and is one of the main causes of DED. The loss of ocular surface homoeostasis may be facilitated by the continuance of this cycle. In order to break the cycle of chronic disease and aid in the restoration of ocular surface equilibrium, considering evaporation to be the fundamental cause of DED indications and manifestation may be helpful. By decreasing tear evaporation, perfluorohexyloctane ophthalmic solution mimics a crucial role of natural meibum.

Previously, the excessive evaporation in DED patients was not addressed and only two of the three main causes of DED—aqueous deficit and inflammation—are targeted by the current standard of prescription care, which includes immunomodulators, anti-inflammatories, and tear stimulators. Remarkably, the cycle of symptoms could continue if the evaporation of tears is not treated directly. For patients suffering from dry eye disease, eye care practitioners can augment their therapeutic arsenal with MIEBO, a potentially effective new treatment option that specifically targets tear evaporation^21^. Also when compared to conventional anti-inflammatory or immunomodulatory treatment, MIEBO improves consistency in blinking and tear film spread by lowering surface tension^19^.

Additionally, as semifluorinated alkanes are known to transport oxygen, perfluorohexyloctane (PFHO) may also have the advantage of acting as an O_2_ carrier like other semifluorinated alkanes. It was discovered that PFHO could carry 62% more O_2_ than air, at a partial pressure of roughly 260 mmHg. Thus, PFHO deposited on the surface of the eye does not act as an oxygen barrier; rather, it helps transport oxygen to the corneal surface, which is essential for preserving the avascular corneal epithelium’s health, encouraging corneal wound healing, and averting corneal oedema^14^.

According to certain theories, perfluorohexyloctane may also promote heat exchange between the environment and corneal tissue, lowering corneal temperature in addition to avoiding evaporation^20^.

In comparison to traditional therapies, Meibo (Perfluorohexyloctane) demonstrates notable advantages. Research indicates that because NOV03 has a refractive index identical to water, it produces fewer visual disruptions after instillation compared to gel or ointment-based therapeutics^1^. Clinical studies have shown mixed results: while the Schirmer without anaesthesia test showed minimal improvement, measures such as TFBUT, OSDI-like research scores, and conjunctival and corneal fluorescein dyeing significantly enhanced^13^. Safety criteria such as BCVA and IOP remained unchanged. Patients undergoing 5–7 weeks of therapy reported reduced symptoms, including burning (40%), fatigued eyes (38%), red eyes (30%), itchiness (26%), blurry vision (23%), and an unusual strange feeling (20%). Moreover, patients generally responded positively to the convenience of administration and the feeling of the drops^13^.

In above mentioned two important safety and efficacy trials, clinical evidence has repeatedly demonstrated that MIEBO dramatically reduces DED symptoms as measured by the visual analogue scale (VAS) dryness score as well as markers such as total corneal fluorescein staining. Furthermore, the strong and well-established tolerability profile of MIEBO increases its potential as a treatment option for DED patients^14^.

As the clinical trials showed that MIEBO improves corneal staining in patients, the researcher is interested in assessing prospective studies that might look into how MIEBO could inhibit friction from mechanical dry eye^19^.

## Conclusion

In conclusion, the review of Meibo (Perfluorohexyloctane) alongside traditional therapies for dry eye disease highlights several key findings. Meibo offers a promising alternative characterized by its unique focus on tear evaporation rather than tear formation. Its formulation, with a refractive index identical to water, minimizes visual disturbances post-application, distinguishing it from gel or ointment-based therapeutics.

Clinical trials have demonstrated significant improvements in objective measures such as tear film breakup time (TFBUT) and ocular surface disease index (OSDI) scores, as well as subjective symptom relief reported by patients. Compared to conventional treatments like artificial tears, cyclosporine, and lifitegrast, Meibo shows competitive efficacy and safety profiles, albeit with specific considerations such as the need for contact lens removal during administration.

Patients have responded positively to the ease of use and perceived comfort of Meibo, reflecting its potential to enhance treatment adherence and patient satisfaction. Looking forward, the future of Meibo in dry eye management appears promising, with ongoing research focusing on expanding its application in various patient populations and exploring long-term efficacy and safety outcomes. However, further studies are needed to address gaps in knowledge, including its use in pregnant, breastfeeding, and paediatric patients.

In clinical practice, Meibo represents a valuable addition to the armamentarium against dry eye disease, offering clinicians a well-tolerated and effective option that targets tear evaporation. Continued investigation and integration into treatment algorithms will be essential to fully realize its potential and optimize patient outcomes in the evolving landscape of ocular surface disorders.

## Ethical approval

Not applicable.

## Consent

Not applicable.

## Source of funding

The authors received no extramural funding for the study.

## Author contribution

The conceptualization was done by B.A. The literature and drafting of the manuscript were conducted by B.A., Y.S., W.U., A.S., Z.U.N.M. and K.A.H.M.A. The editing and supervision were performed by B.A. and injunction. All authors have read and agreed to the final version of the manuscript.

## Conflicts of interest disclosure

The authors declare no potential conflicts of interest concerning the research, authorship, and/or publication of this article.

## Research registration unique identifying number (UIN)

1. Name of the registry: Not required because this is an editorial.
2. Unique Identifying number or registration ID: Not applicable because this is an editorial.
3. Hyperlink to your specific registration (must be publicly accessible and will be checked).

## Guarantor

Dr Khabab Abbasher Hussien Mohamed Ahmed.

## Data availability statement

Data availability is not applicable to this article as no new data were created or analyzed in this study.

## Provenance and peer review

Not commissioned, externally peer-reviewed.
